# Optimisation of the Adhesion of Polypropylene-Based Materials during Extrusion-Based Additive Manufacturing

**DOI:** 10.3390/polym10050490

**Published:** 2018-05-02

**Authors:** Martin Spoerk, Joamin Gonzalez-Gutierrez, Christof Lichal, Hrvoje Cajner, Gerald Roman Berger, Stephan Schuschnigg, Ludwig Cardon, Clemens Holzer

**Affiliations:** 1 Polymer Processing, Montanuniversitaet Leoben, Otto Gloeckel-Straße 2, 8700 Leoben, Austria; joamin.gonzalez-gutierrez@unileoben.ac.at (J.G.-G.); christof.lichal@unileoben.ac.at (C.L.); stephan.schuschnigg@unileoben.ac.at (S.S.); clemens.holzer@unileoben.ac.at (C.H.); 2 Centre for Polymer and Material Technologies, Department of Materials, Textiles and Chemical Engineering, Ghent University, Technologiepark 915, 9052 Zwijnaarde, Belgium; ludwig.cardon@ugent.be; 3 Faculty of Mechanical Engineering and Naval Architecture, University of Zagreb, Ivana Lučića 1, Zagreb 10002, Croatia; hrvoje.cajner@fsb.hr; 4 Injection Moulding of Polymers, Montanuniversitaet Leoben, Otto Gloeckel-Straße 2, 8700 Leoben, Austria; gerald.berger@unileoben.ac.at

**Keywords:** additive manufacturing, fused filament fabrication, adhesion, polypropylene, ultra-high-molecular-weight polyethylene, surface roughness, parametric study

## Abstract

Polypropylene (PP) parts produced by means of extrusion-based additive manufacturing, also known as fused filament fabrication, are prone to detaching from the build platform due to their strong tendency to shrink and warp. Apart from incorporating high volume fractions of fillers, one approach to mitigate this issue is to improve the adhesion between the first deposited layer and the build platform. However, a major challenge for PP is the lack of adhesion on standard platform materials, as well as a high risk of welding on PP-based platform materials. This study reports the material selection of build platform alternatives based on contact angle measurements. The adhesion forces, investigated by shear-off measurements, between PP-based filaments and the most promising platform material, an ultra-high-molecular-weight polyethylene (UHMW-PE), were optimised by a thorough parametric study. Higher adhesion forces were measured by increasing the platform and extrusion temperatures, increasing the flow rate and decreasing the thickness of the first layer. Apart from changes in printer settings, an increased surface roughness of the UHMW-PE platform led to a sufficient, weld-free adhesion for large-area parts of PP-based filaments, due to improved wetting, mechanical interlockings, and an increased surface area between the two materials in contact.

## 1. Introduction

Fused filament fabrication (FFF), also known as fused deposition modelling (FDM^TM^), is the most common type of extrusion-based additive manufacturing or material extrusion [1]. The technique is based on the selective deposition of thermoplastic filaments, which are transported by counter-rotating driving wheels through a nozzle that moves according to a predefined contour [2]. Complex three-dimensional parts are shaped by a layer-by-layer deposition of the liquefied material onto a build platform [3]. The equipment used in FFF is relatively inexpensive, compared to other additive manufacturing technologies, and is safe and simple to operate [4]. All of these reasons have contributed to the increased popularity of FFF. Its main advantage is the rapid and economical reproduction of customised components without design constraints with a variety of polymeric materials [5]. 

Recently, the fabrication of polypropylene (PP)-based materials by means of FFF has increasingly attracted attention [6,7,8,9,10,11,12,13,14,15,16,17,18,19], due to its low cost, low density, high impact strength, improved chemical resistance, and its higher potential to substitute engineering materials, compared to the standard filament materials used for FFF [20]. It has been shown that the main drawback of PP of having a high tendency to warp can be mitigated by the incorporation of fillers [6,8,9,11,16], and by a dexterous selection of process parameters [12,19]. Another possibility to control the warpage of 3D-printed PP, especially for unfilled PP, is to maximise the adhesion of the first deposited layer to the build platform of FFF machines, as the adhesion forces counteract the forces that pull the deposited parts away from the surface [21]. However, an issue of PP is the lack of adhesion to traditional platform materials [10], which can result in an early delamination of the first layer [22]. Consequently, the previously deposited layers do not stay in place during the build cycle, which can have detrimental effects on part quality [6], and eventually on the mechanical properties of the produced parts [23,24]. So far, studies on FFF with PP either do not mention the platform material [7,13,15,25], or try to overcome this issue by depositing PP onto PP-tapes [6,17] or PP-plates [8,9,11,12,16]. Nevertheless, printing on the same material as the extruded filament can easily lead to welding of the built part onto the platform [12], which causes difficulties during the removal of the part. Due to the welding of PP onto PP, typical strategies to improve adhesion, such as increasing the platform [21,22] or nozzle temperature [26], or decreasing the thickness of the first deposited layer [27], are not applicable. Moreover, the approach of printing directly above the glass transition temperature (T_G_) of the filament to obtain highest possible adhesion, as has been found for poly(lactic acid) (PLA) and acrylonitrile butadiene styrene (ABS) [21], is inapplicable for PP due to its low T_G_ [28]. Therefore, a novel approach for maximising the adhesion of PP during FFF is desirable. 

The present study attempts to close this gap by elucidating the material selection of a novel build platform material for PP-based materials. Therefore, a material is sought that exhibits a similar chemical composition, but marginally different molecular structure, compared to the filament material, in order to achieve slightly different interfacial tensions between the materials that are in contact. The optimisation of adhesion forces between the most promising platform material and a PP-based filament obtained by a shear-off measurement device is presented through a thorough parametric study, and by evaluating the influence of the surface roughness and the temperature of the platform material. The systematic approach of the present work provides the basis of flawless printability of this versatile material, whilst counteracting its tendency to warp and preventing welding of the contact pairs. 

## 2. Materials and Methods

### 2.1. Materials

Unless stated otherwise, for all prints in this work, a PP filament optimised for improved warpage control (PP-composite) that consisted of 61.2 vol. % of a PP random copolymer (rPP, Borealis AG, Wien, Austria) with an average molecular weight of 2.4 × 10^5^ g·mol^−1^, a polydispersity index of 4.3 and a melt flow rate of 8 g/10 min (230 °C/2.16 kg), 2.0 vol. % of the compatibiliser SCONA TPPP 9212 GA (BYK-Chemie GmbH, Wesel, Germany), 6.8 vol. % of the amorphous polyolefin Aerafin 180 (Eastman Chemical Company, Kingsport, TN, USA), and 30 vol. % of perlite fillers (Bublon GmbH, Gleisdorf, Austria) was used. The PP-composite was prepared by melt-compounding in a kneader (Polylab Rheomix 3000, Thermo Fisher Scientific Inc., Waltham, MA, USA). More details about its preparation are given in Ref. [8]. Two build platform materials were tested: An extruded PP heterophasic copolymer (hPP, average molecular weight of 2.9 × 10^5^ g·mol^−1^, a polydispersity index of 4.5, and a melt flow rate of 5 g/10 min (230 °C/2.16 kg)) plate with dimensions of 600 × 400 × 10 mm^3^ was supplied by AGRU Kunststofftechnik GmbH, Austria. The ultra-high-molecular-weight polyethylene (UHMW-PE) plate ISO-LEN^®^ 1000 natur with a size of 600 × 400 × 7 mm^3^ and a comparable surface roughness to hPP was purchased from Iso-Tech Kunststoff GmbH, Germany. For the material preselection, the aforementioned materials were compared to a PLA filament and a glass mirror, both obtained from Prirevo e.U., Austria. For the final verification of the usability of the UHMW-PE as a build platform, apart from the PP-composite, neat rPP [8], the hPP [9], and the hPP filled with carbon fibres [11] were used as filament materials. All filaments used in the present work were prepared using the filter test single screw extruder FT-E20T-MP-IS (Dr. Collin GmbH, Ebersberg, Germany) set to 60 rpm and 185 °C, equipped with a die of 1.9 mm in diameter and 25.05 mm in length. The extrudate was cooled in a 3 m long water-bath set to roughly 50 °C, and was pulled and spooled by a winding unit. 

### 2.2. Contact Angle Measurements

To preselect possible platform materials that exhibit a sufficient adhesion to PP, contact angle measurements were performed with a Krüss DSA100 (Krüss GmbH, Hamburg, Germany) at room temperature. As platform materials, the glass mirror, rPP, hPP, and UHMW-PE, and as filament materials the PLA, rPP, and PP-composite were investigated. All materials that had not been purchased as plates (rPP, PLA, and PP-composite) were pressed to plates in a Collin P200PV vacuum press (Dr. Collin GmbH, Germany) at 200 °C and 15 MPa for 25 min with the same mould, to obtain a similar surface roughness. This was done to be able to make a direct comparison of the contact angle measurements, which are influenced by the surface roughness [29]. The contact angles between each of these materials and the test-liquids (deionised water, diiodomethane, and ethylene glycol) were measured. Per combination, fifteen repetitions were performed, considering the propagation of uncertainty. The results were evaluated as interfacial tensions as described in Ref. [30,31,32]. The interfacial tension $\sigma_{int}$ was calculated as:

(1) $$\sigma_{int}=\;\sigma_{s}^{P}+\sigma_{s}^{D}+\sigma_{l}^{P}+\sigma_{l}^{D}-2\cdot \left(\;\sqrt{\sigma_{s}^{D}\cdot \sigma_{l}^{D}}+\sqrt{\sigma_{s}^{P}\cdot \sigma_{l}^{P}}\right)$$

in which $\sigma_{l}^{P}$ and $\sigma_{l}^{D}$ are the known polar and disperse fractions of the surface tension of the test liquids, respectively, and $\sigma_{s}^{P}$ and $\sigma_{s}^{D}$ are the polar and disperse fractions of the surface energy of the investigated polymers, respectively. $\sigma_{s}^{P}$ and $\sigma_{s}^{D}$ are calculated as the slope and the axis intercept, respectively, for a fitted line for the three test liquids of Equation (2), in which Θ is the mean of the 15 investigated contact angles.

(2) $$\frac{\left(1+\cos\Theta \right)\cdot \left(\sigma_{l}^{P}+\sigma_{l}^{D}\right)}{2\cdot \sqrt{\sigma_{l}^{D}}}\;=\;\sqrt{\sigma_{s}^{P}}\cdot \sqrt{\frac{\sigma_{l}^{P}}{\sigma_{l}^{D}}}+\sqrt{\sigma_{s}^{D}}$$

### 2.3. Printer Settings

All prints were performed on a Hage 3DpA2 (Hage Sondermaschinenbau GmbH & Co. KG, Obdach, Austria) equipped with a brass nozzle of 0.5 mm in diameter. A printing speed of 50 mm·min^−1^ was kept constant for all specimens. Apart from the parameter changes in the design of experiments (DOE, Section 2.5), all prints were conducted with a constant nozzle temperature of 200 °C and an extrudate flow rate of 2 mm^3^·s^−1^. Those build platforms that were heated up beyond room temperature were glued by employing the instant adhesive “Sekundenkleber hitzebeständig” and a primer (both Toolcraft, Conrad Electronic International GmbH & Co. KG, Hirschau, Germany) onto a stainless steel plate with a thickness of 5 mm, in order to prevent warpage of the polymeric plate. Only this set-up guaranteed a perfectly levelled platform, which was a requirement for the subsequent adhesion measurements. To prevent changes in the thickness of the polymeric platform due to its thermal expansion, the whole build platform was levelled precisely before each build cycle so that the distance between the nozzle and the platform was constant at every point on the platform. Within one print, 10 separate strands, 8 mm apart from each other, were printed, of which each strand had a length of 100 mm and a height of 0.4 mm, consisting of two layers. The width of the strand depended on the thickness of the first layer and the platform temperature used. 

### 2.4. Adhesion Measurement

**Figure 1:**
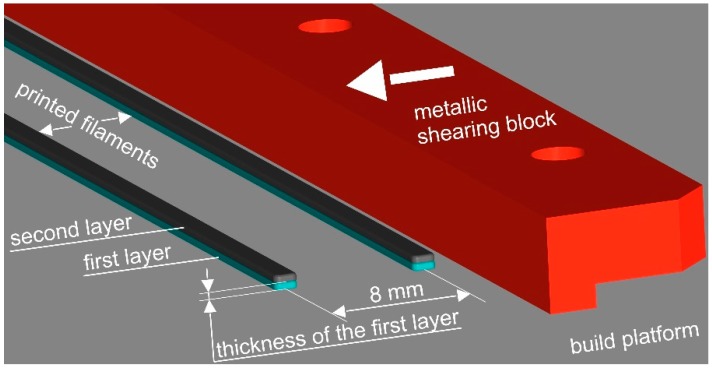
Schematic representation of the shear-off testing mechanism of the printed filaments by a metallic shearing block, along with a description of the most important expressions used throughout the manuscript. The arrow on the metallic shearing block symbolises its horizontal movement that causes the detachment of the filaments from the build platform.

The adhesion forces between printed strands and the build platform were characterised by means of a self-developed shear-off force testing device. A detailed explanation of the instrumentation and the measurement technique is given in Ref. [21]. In brief, the metallic shearing block, which had a distance of approximately 0.1 mm to the build platform over the whole measurement area, moved horizontally over the build platform at a speed of 2 mm·s^−1^ to shear-off a single printed strand, which is oriented parallel to the shearing block (Figure 1). After stopping the horizontal movement and the manual removal of the sheared-off strand, the subsequent strand was tested. This methodology was repeated until all 10 separate strands per setting were measured. All tests were performed in a room under standardised ambient conditions (23 °C, 50% relative humidity). The adhesion forces of each strand and the displacement of the shearing block were determined by the miniature load cell U9C 1 kN and the inductive displacement transducer W100, respectively, through a Spider 8 Data Acquisition System at 300 Hz and the software CatmanAP V3.5.1 (all Hottinger Baldwin Messtechnik GmbH, Darmstadt, Germany). Additional details are explained in Ref. [21]. No cleaning agent was used to clean the build platform in order not to chemically degrade the polymer surface. Instead, dry paper was used. All adhesion force maxima per setting were evaluated to a significance level of 5%. 

In a previous investigation [21], it was found that measuring a shear-off force larger than 200 N for PLA and ABS on a glass surface or polyimide tape can be related to the production of parts with low warpage and without detachment from the build platform, at similar conditions to the ones used during shear-off testing. Therefore, in the present work, a force of 200 N was set as the threshold, beyond which the adhesion of PP-based filaments is sufficient to prevent detachment from the build platforms investigated. On the other hand, measuring shear-off forces larger than 1000 N at the printing conditions for PLA and ABS might indicate that the deposited strands welded to the build platform, as such large forces cannot be measured by the shear-off device used in this investigation. Thus, the acceptable range of shear-off forces should be above 200 N and below 1000 N at the selected printing conditions for PP and the build platforms. This range of forces is an empirical value applicable at all temperatures and layer thicknesses, but only if a similar methodology is used to deposit the strands on the build platform and to shear them off later on.

### 2.5. Statistical Modelling and Optimisation

In a separate two-factorial DOE, the adhesion forces between the PP-composite and a UHMW-PE platform set to 50 °C were modelled and optimised. The methodology was performed using the software Design Expert 10.0 (Stat-Ease, Inc., Minneapolis, MN, USA). The process factors and their levels are summarised in Table 1. The test order was completely randomised and all design points were replicated once. Each design point consisted of 10 measured strands. The analysis of significance was performed by means of the analysis of variance (ANOVA) method with a significance level of 5%. 

### 2.6. Modification of the Build Platform

To further increase the adhesion between the PP-composite and the UHMW-PE platform, the UHMW-PE surface was modified by sanding it to different roughness levels. Sanding was done using the scrub sponge Scotch-Brite^TM^ heavy duty (3M Co., Flemington, NJ, USA), as well as sandpapers with an average particle diameter of 82 µm (grit size of 180) and 190 µm (grit size of 80). They were individually applied under a constant force onto the unmodified UHMW-PE without a predominant sanding orientation for 30 s. The modified plates were used in the same way as the unmodified one (Section 2.3).

### 2.7. Surface Roughness Characterisation

All UHMW-PE surfaces were characterised by means of the Infinite Focus (Alicona Imaging GmbH, Raaba, Austria) 3D-microcoordinate measurement system to identify the surface roughness. To comply with the standards ISO 4287 and ISO 4288 for the surface characterisation measurements, the sanding papers were applied with a distinct orientation on separate smaller UHMW-PE plates with the same sanding level as described in Section 2.6. For the unmodified surface and the one that was treated by the scrub sponge, a 20-fold magnification was used, which resulted in a vertical and lateral resolution of 50 nm and 1.5 µm, respectively. The surface roughness was evaluated over a measurement length of 5.6 mm with a threshold wavelength of 800 µm. The two remaining sanded surfaces were characterised by a 10-fold magnification, resulting in a vertical and lateral resolution of 100 nm and 3 µm, respectively. The surface roughness was evaluated as described above, but with a threshold wavelength of 2500 µm. The visualisation of the surface topography of each surface was done under the same settings, but in a smaller area of 710 × 538 µm^2^. 

### 2.8. Microscopy

The contact surface of one printed strand per UHMW-PE surface modification was analysed in the optical microscope Olympus SZX12 (Olympus Life Science Europe GmbH, Hamburg, Germany), at a magnification of 50× under reflected light, directly after testing their adhesion forces.

## 3. Results and Discussion

### 3.1. Adhesion of the Polypropylene (PP)-Composite on hPP

**Figure 2:**
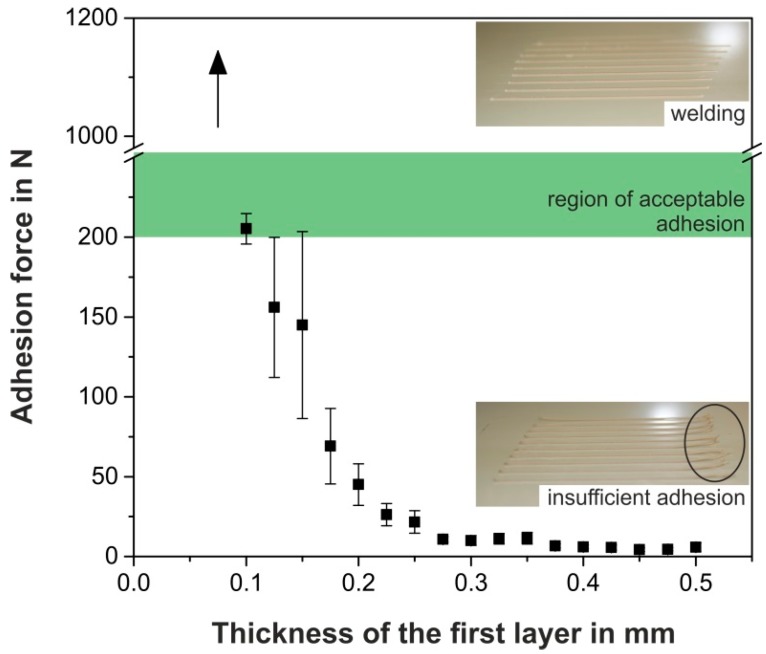
The adhesion force between the printed polypropylene (PP)-composite and the PP heterophasic copolymer (hPP) build platform as a function of the thickness of the first layer measured at room temperature. The arrow in the top left corresponds to overshot forces due to welding. In the top right, the undesired welding of the two materials at thicknesses of the first layer below 0.1 mm is represented. The region of acceptable adhesion according to Ref. [21] is marked in green. In the bottom right, the warping of the strands, as highlighted by the circle, due to insufficient adhesion is illustrated for thicknesses of the first layer that are higher than 0.2 mm.

It is evident for FFF that the strategy of printing PP-based filaments onto PP build platforms implies the risk of welding between the two contact partners [33,34], due to their comparable polar and disperse fractions [30]. A similar finding was discovered for the adhesion between the deposited PP-composite and the hPP build platform at room temperature (Figure 2). Although the addition of 30 vol. % of mineral fillers generally decreases the weld strength [35,36] and alters the surface energy [10], strong welds were found for a thickness of the first layer below 0.1 mm. These welded strands led to adhesion forces that overshot the maximum load of the force transducer (>1000 N, marked as an arrow in Figure 2) in the self-developed shear-off force testing device. In fact, parts printed at these settings reveal very good adhesion to the build platform; so good that they cannot be removed without damaging the part, the platform, or both. Hence, welding during the deposition of the first layer in FFF should be avoided. This can be achieved by increasing the thickness of the first layer [27]. As the adhesion forces decrease rapidly with rising thicknesses of the first layer (Figure 2), similarly to Ref. [27], and as explained in Section 2.4, adhesion forces of approximately 200 N are recommended for a reliable print [21], only a very narrow processing window is found (around a thickness of 0.1 mm for the first layer) in which an acceptable level of adhesion is given (marked in green in Figure 2). Thicknesses of the first layer above 0.2 mm, which are common for standard FFF materials [2], already result in extensive warpage due to insufficient adhesion, as highlighted in the inset of Figure 2. 

For an ideal warpage control of PP parts produced by FFF, an additional increase in the printing chamber temperature, mostly controlled by augmenting the platform temperature, is essential [12]. However, welding at higher thicknesses of the first layer can be expected for elevated platform temperatures, as higher weld strengths have been observed for increased mould temperatures for the weld lines of injection-moulded PP [37]. Hence, the processing window for acceptable adhesion is further reduced. Ideal settings at a high temperature platform would allow sufficient adhesion to build a part without welding, and damage-free removal at room temperature, as was observed for PLA and ABS [21]. However, in the present case, these settings can hardly be provided. Hence, PP build platforms cannot be fully recommended for PP-based filament materials, especially not for reliable, flawless prints, in which process parameters should be adaptable for different geometries. 

### 3.2. Build Platform Material Selection for PP-Based Filaments

In order to preselect an appropriate build platform alternative, the surfaces of various material combinations were investigated by means of contact angle measurements (Section 2.2). To determine the compatibility between the filament materials and possible build platforms, the interfacial tensions, which are dependent on the polarities and the surface energies of the materials in contact [30], and are inversely proportional to their adhesion [30,38], are calculated based on the contact angles between the test-liquids and the surfaces [30,39,40]. 

**Figure 3:**
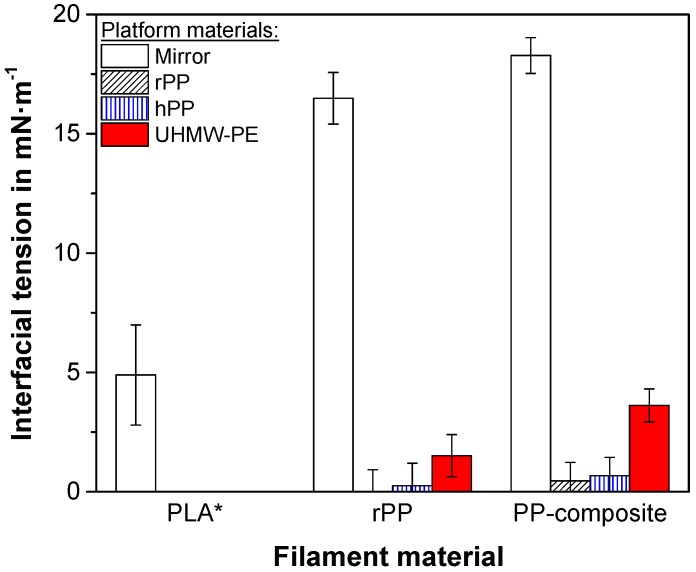
Interfacial tension between the four platform material options and the filaments investigated. The interfacial tension for poly(lactic acid) (PLA) on the mirror, assigned by an asterisk, was measured for the settings in which ideal adhesion was achieved. Hence, it represents a measurement for a platform temperature of 70 °C, taken from Ref. [21], and serves as a reference value for the other material combinations. All other values were obtained at room temperature.

In order to have a reference value for a proper adhesion during FFF, the interfacial tension for the standard FFF filament material PLA and its most promising build platform, a glass mirror, at a platform temperature of 70 °C, at which the highest adhesion forces were found [21], is given in Figure 3. Considerably higher interfacial tensions than that of PLA (4.9 ± 2.1 mN·m^−1^) are not desirable, as this could result in a lack of adhesion, whereas significantly lower interfacial tensions show the tendency of too good adhesion, and therefore the possible formation of welds [41]. The rather large errors in the interfacial tensions result from the susceptibility to contact angle deviations due to external influences, e.g., temperature or roughness [31]. Hence, an ideal adhesion can be expected at the mean interfacial tension of PLA. 

The unfilled rPP as a filament material shows a very high interfacial tension in contact with the glass mirror (16.5 ± 1.1 mN·m^−1^, Figure 3), which confirms the non-existent adhesion of printed rPP on the mirror. As expected, the interfacial tension is minimal (~0 mN·m^−1^), equalling a maximum adhesion, if the filament and the build platform are made of the same material (e.g., rPP on rPP). As a result, this combination resulted in extensive welding, similar to Figure 2. It is known from literature [12] that slight changes in the PP-type of the build platform, such as a change from rPP to hPP, can decrease the risk of welding. A similar trend can be confirmed by a slightly increased interfacial tension to 0.3 ± 0.9 mN·m^−1^ (Figure 3). However, this alteration is insignificant and results in an interfacial tension that is far from the ideal one found for PLA on the glass mirror. Therefore, bigger differentiations, other than the molecular structure of the PP-types, are necessary. One possible material solution that is similar in terms of chemical composition, but different enough in terms of the morphology, is UHMW-PE [42]. Combined with the rPP filament, the UHMW-PE plate reveals a clearly increased interfacial tension (1.5 ± 0.9 mN·m^−1^), which corresponds to a trend towards the desired direction of the reference value. This hints at performing better as a build platform than any PP-plate. 

If the PP-composite is used as the filament material (Figure 3), the interfacial tensions for the platform materials investigated show the same trends as for the unfilled rPP filament. Nevertheless, the addition of the mineral filler causes an overall increase in the interfacial tension, similarly to Ref. [10], as the adhesion decreases due to the filler. This trend is in agreement with studies on the weld strength of filled PP [35]. For the PP-composite, the adhesion to the UHMW-PE surface seems to be promising, since the interfacial tension of this combination (3.6 ± 0.7 mN·m^−1^) is comparable to that of the guiding value of PLA. As the UHMW-PE is expected to be an ideal substitution for PP-plates, the following investigations are conducted on this surface only. 

### 3.3. Adhesion of the PP-Composite on Ultra-High-Molecular-Weight Polyethylene (UHMW-PE)

#### 3.3.1. Effect of the Platform Temperature

**Figure 4:**
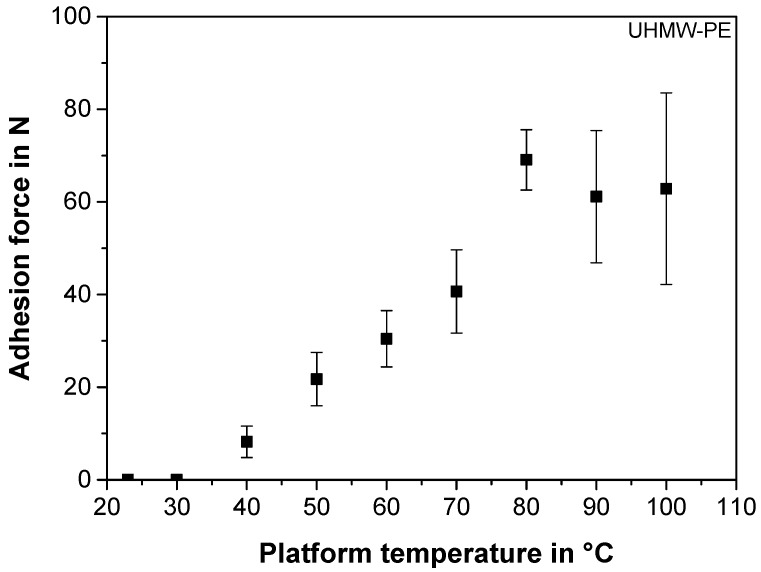
The adhesion force between the printed PP-composite and the ultra-high-molecular-weight polyethylene (UHMW-PE) build platform as a function of the platform temperature, for a constant thickness of the first layer of 0.2 mm.

To investigate the adhesion forces as a function of the UHMW-PE platform temperature, without having considerable influences from local height alterations of the plate at higher temperatures, the thickness of the first layer was increased to 0.2 mm for the following investigation. Similarly to 3D-printed PLA and ABS [21] and polyetheretherketone [22], as well as to investigations on the weld lines of PP during injection moulding [37], the PP-composite shows increasing adhesion forces obtained from the shear-off force testing device (Section 2.4) for rising platform temperatures, when printed onto the UHMW-PE platform (Figure 4), as the polymer chain mobility of both materials in contact increases with temperature [43]. As expected from the significantly higher interfacial tension between the filament and the UHMW-PE, compared to that of the filament and any PP plate (Figure 3), no welding occurs between the two materials for any temperatures investigated. Moreover, at room temperature, the PP-composite does not adhere to the UHMW-PE, which is essential for the damage-free removal of the part [21]. In contrast, the same settings for the prints onto the hPP plate at room temperature reveal a considerably higher adhesion force of 45.0 ± 13.0 N (Figure 2), which confirms the suitability of the UHMW-PE as a platform material. 

#### 3.3.2. Adhesion Optimisation by a Parametric Study

As adhesion forces of around 70 N are far from the recommended 200 N [21], and therefore insufficient for a reliable print (Figure 4), the adhesion between the PP-composite and the UHMW-PE is optimised by a DOE, with limits shown in Table 1. For platform temperatures beyond 50 °C, the UHMW-PE exhibited slight local height alterations, due to thermal expansion, and a visible amount of warpage of the plate. Since the adhesion shear-off measurements are sensitive to such changes, the experiments for the DOE are investigated for a constant platform temperature of 50 °C in order to gain reliable and repeatable results. The adhesion forces for all investigated shear-off measurements in the DOE are summarised in Table 2. 

**Figure 5:**
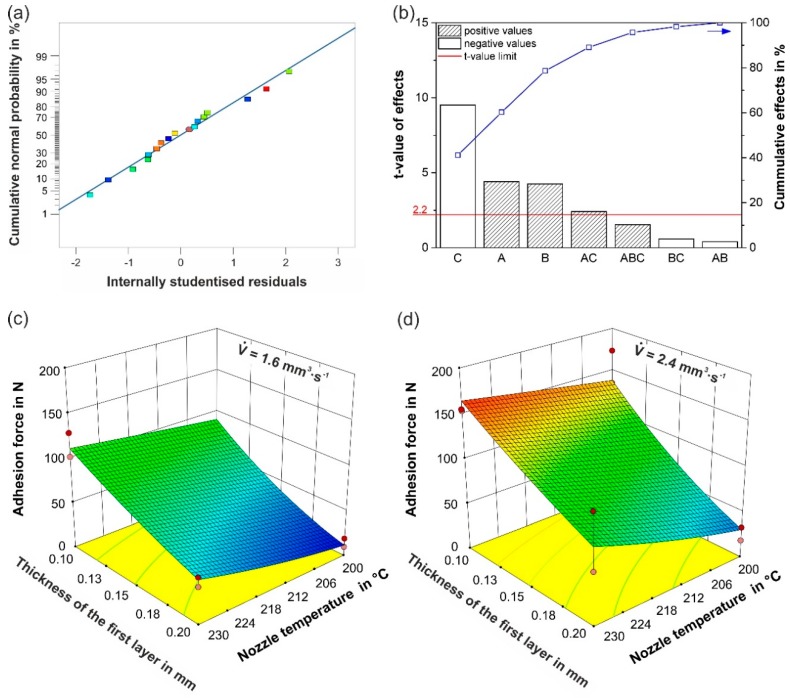
The normal probability plot for all tested values (**a**), the Pareto chart for the effects obtained by the ANOVA (**b**), and the regression function plots for flow rates of 1.6 mm^3^·s^−1^ (**c**) and 2.4 mm^3^·s^−1^ (**d**). In (**b**), the designations A, B, and C refer to the nozzle temperature, the flow rate, and the thickness of the first layer, respectively. The horizontal line in (**b**) represents the *t*-value limit, which determines the significance limit, and the filling pattern of the bars reveals the effect direction. A positive value, for example, means that the adhesion force increases, when the predictor variables or their interactions change from their lower to their higher setting. In (**c**,**d**), red/orange circles represent the mean values of each of the two replications. These can be below (orange) or above (red) the regression surface.

For the statistical modelling, the square root transformation guaranteed normally distributed residuals (Figure 5a) and homoscedasticity of variance. As a result of the ANOVA, the Pareto chart (Figure 5b) shows the significant effects. As expected from Figure 2, the thickness of the first layer (C) reveals the biggest effect (*t*-value of 9.52) on the adhesion force (F_ad_), in the negative direction. This means that a reduction of C from 0.2 to 0.1 mm (Table 1) leads to the strongest increase of the adhesion forces. The nozzle temperature (A) and the flow rate (B) exhibit comparable, significant effects on the adhesion force, both in the positive direction. Hence, increasing A from 200 to 230 °C or B from 1.6 to 2.4 mm^3^·s^−1^ causes significantly higher adhesion forces. Additionally, the effect of the interaction AC cannot be neglected. These trends are in accordance with investigations on 3D-printed polymers onto textiles [26], and on injection-moulded weld lines for PP, in which higher weld strengths were found for higher melt temperatures [37], which corresponds to an increase in A, and higher holding pressures [44], which could resemble an increase in B or a decrease in C. The regression equation for the process factors is

(3) $$F_{\mathrm{ad}}{}^{0.5}=21.22-0.05A+2.79B-231.17C+0.84\mathrm{AC}$$

and the model is presented as response surface plots together with the measured data in Figure 5c for B of 1.6 mm^3^·s^−1^, and in Figure 5d for B of 2.4 mm^3^·s^−1^. The model seems to be precise, as it shows a suitable distribution of the residuals (Figure 5a), a high signal to noise ratio of 16.3, and a relatively high coefficient of determination R^2^ of 0.90. 

In contrast to the hPP build platform, and as expected from Figure 4, none of the investigated settings resulted in welding between the PP-composite and the UHMW-PE, in spite of the low thickness of the first layer investigated. According to the DOE, the setting for optimal adhesion is found to be at the higher nozzle temperature of 230 °C, the higher flow rate of 2.4 mm^3^·s^−1^, and the lower thickness of the first layer of 0.1 mm. With this setting, a maximum adhesion force of 155.5 ± 11.2 N (mean for both replications) is achieved. As this force is still below 200 N, it can be considered as insufficient for reliable large-area prints [21]. As a result, two further strategies can be applied. Firstly, the current platform temperature of 50 °C can be further increased to improve the chain mobility of the partners in contact (compare to Figure 4). This is a very practical solution that can also be easily applied to larger parts. However, the characterisation of the actual adhesion forces by means of the shear-off device is not applicable at temperatures above 50 °C, due to the uneven thermal expansion of the polymeric plate. Another approach to improve the adhesion forces of the first deposited layer is to alter the roughness of the platform material, since it has a considerable effect on the interfacial tension [31,32], the mechanical interlocking [27,39,45], and therefore on adhesion [39].

#### 3.3.3. Effect of Surface Roughness

**Figure 6:**
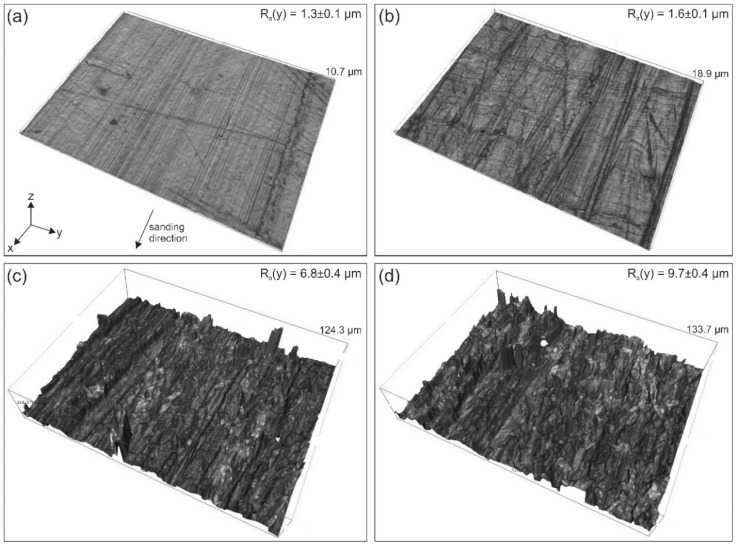
Topography images of the UHMW-PE building platform: Unmodified (**a**); sanded by a scrub sponge (**b**); by sandpaper with an average particle diameter of 82 µm (**c**); and by sandpaper with an average particle diameter of 190 µm (**d**). All images show an area of 710 × 538 µm^2^. The coordinate system in (**a**) represents the specimen orientation for all scanned samples. The sanding orientation is in the x-direction, as highlighted in (**a**). In the top right corners, the corresponding mean and standard deviation of the surface roughness (R_a_(y)), measured in the y-direction, are shown. The maximum height of the measured profile is presented next to each topography image.

The sanding procedure (Section 2.6) drastically changes the topography of the UHMW-PE (Figure 6). For example, the surface roughness R_a_(y) increases from 1.3 ± 0.1 µm to 9.7 ± 0.4 µm, and height irregularities of up to 130 µm are introduced with increasing sanding particle diameter. The latter suggest the possibility of mechanical interlockings between the strands and the substrate that can influence the adhesion forces [27,39,45]. Results from the literature [29,46,47,48,49,50] suggest that an increased surface roughness is important to modify the wettability of a liquid onto that surface. Depending on the pattern achieved by the roughening mechanisms, and the amount of roughness obtained, the wetting can either increase [29,47,49,50,51] or decrease [29,48,49]. 

**Figure 7:**
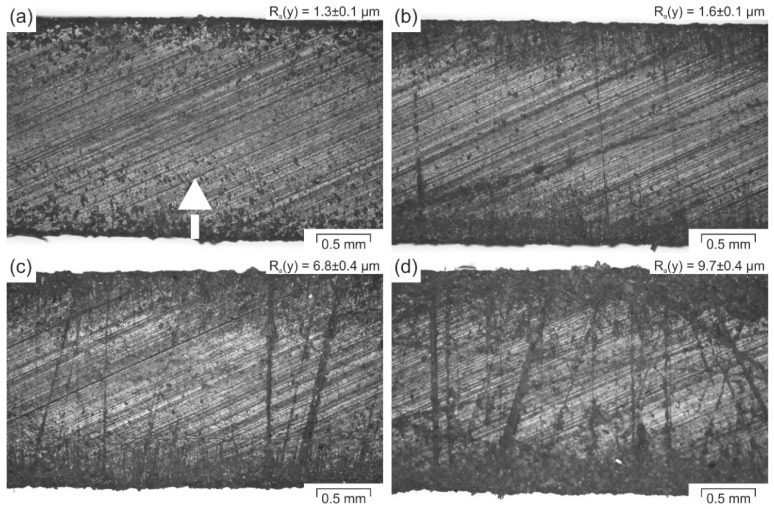
Optical microscopy images of a strand of the printed PP-compound after being sheared-off from differently modified UHMW-PE build platform surfaces: Unmodified (**a**); sanded by a scrub sponge (**b**); by sandpaper with an average particle diameter of 82 µm (**c**); and by sandpaper with an average particle diameter of 190 µm (**d**). In the top right corner of each image, the corresponding R_a_(y)-value of the build platform, onto which the strand was printed, is represented. The arrow displayed in (**a**) highlights the loading direction of the measuring device, which is the same for all four images.

It appears that the pattern and level of roughness achieved in the present investigation lead to a better wetting of the molten PP on the rougher UHMW-PE. This can be seen in the areas of the printed strands that are in contact with the differently modified UHMW-PE surfaces, by more prominent marks on the surface for rising roughness (Figure 7), as the melt seems to effectively wet the irregular surfaces. 

**Figure 8:**
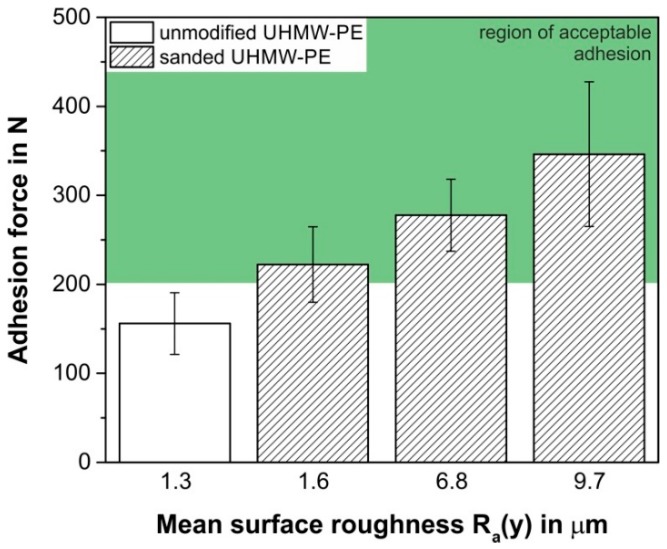
Adhesion forces of the PP-composite as a function of the arithmetic mean surface roughness of the UHMW-PE build platform. All results were obtained under optimised processing conditions, which represent the maximum of the regression function plot of Figure 5d.

Consequently, mechanical interlockings are formed, which cause a significant increase in the adhesion forces (more than 100%) for a higher surface roughness, compared to the unsanded substrate (Figure 8). Additionally, the rougher surface exhibits an increased contact area [51] and therefore favours more diffusion across the interface to the PP-composite, which leads to enhanced adhesion forces [21]. As a result, the optimised printing settings and the highest applied surface roughness yield adhesion forces of 346.2 ± 81.3 N, while no welding occurs. 

**Figure 9:**
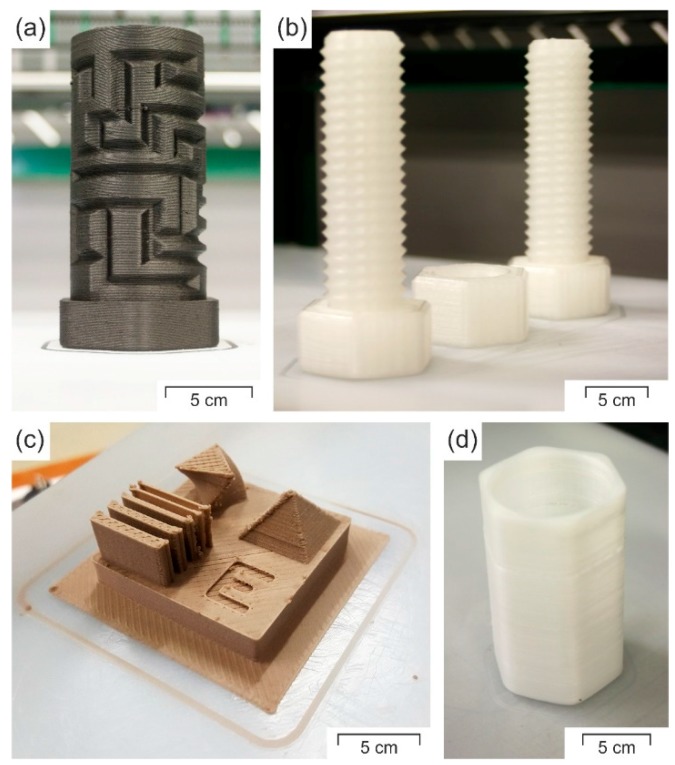
Representation of the practical usability of the sanded UHMW-PE plate as a build platform material for PP-based materials: hPP filled with carbon fibres [11] (**a**); unfilled PP random copolymer (rPP) [8] (**b**); PP-composite [8] (**c**); and neat hPP [9] (**d**). All printed specimens are represented directly after the finalisation of the print, when still in contact with the UHMW-PE build platform.

These high forces are sufficient for reliable prints of different sizes. This is confirmed for various PP-based filament materials in Figure 9, in which technical parts are represented directly after the completion of the print, still in contact with the UHMW-PE build platform. After the removal of the parts, the area that was in contact with the rougher build platform exhibits a rougher surface, due to wetting (Figure 7). However, this roughness is in the microscale, and therefore is insignificant compared to the roughness induced by the strands [52,53]. Additionally, Figure 9 confirms that none of the depicted parts exhibit extensive warpage due to the strong adhesion of the first deposited layer, although the unfilled materials were especially shown to be prone to warpage when printed onto PP-surfaces [8,12]. Nonetheless, large-area parts that are very prone to warpage could still detach from the platform, despite the optimised settings. If so, an increase in the build platform temperature above 50 °C should mitigate this issue. 

## 4. Conclusions

In summary, the present study describes the welding problem of 3D-printed PP-based filament materials on PP-surfaces, and concurrently suggests an ideal substitution of the PP build platform by UHMW-PE plates. These were selected based on a similar interfacial tension compared to the standard filament material PLA. The adhesion of deposited PP strands on the UHMW-PE, characterised by shear-off force measurements, was optimised by a thorough parametric study. Whilst preventing the welding of the first deposited layer with the UHMW-PE surface and, hence, guaranteeing a damage-free removal of the final part at room temperature, highest adhesion forces were obtained for high platform temperatures, high nozzle temperatures, high flow rates, and low thicknesses of the first layer. An increased surface roughness of the UHMW-PE plate can lead to mechanical interlockings and a larger contact area between the printing material and the platform, due to improved wetting. This resulted in adhesion forces that are sufficient to prevent the detachment of the printed part from the platform and, hence, the warpage in large-area prints for a variety of PP-based filament materials. The systematic material selection by means of contact angle measurements, the printing optimisation, and the surface roughness enhancement presented in this study could be readily applied to avoid the detachment of the first layer of other semi-crystalline thermoplastics produced by extrusion-based additive manufacturing, especially to materials that are prone to warpage or to novel polymer composites. 

## Figures and Tables

**Table 1:** The investigated factors and their corresponding designation and low and high level of the design of experiments (DOE).

Designation	Factors	Low Level	High Level
A	Nozzle temperature (°C)	200	230
B	Flow rate (mm^3^·s^−1^)	1.6	2.4
C	Thickness of the first layer (mm)	0.1	0.2

**Table 2:** Summary of all adhesion force results for the parametric investigation of the DOE.

Factors	Adhesion Forces (N)							
A (°C)	200	200	230	230	200	200	230	230
B (mm^3^·s^−1^)	1.6	1.6	1.6	1.6	2.4	2.4	2.4	2.4
C (mm)	0.1	0.2	0.1	0.2	0.1	0.2	0.1	0.2
Replication 1	73.9 ± 25.4	33.8 ± 6.5	101.3 ± 13.6	40.2 ± 10.2	174.3 ± 18.8	19.5 ± 6.4	154.6 ± 20.3	57.8 ± 6.3
Replication 2	62.0 ± 26.8	18.8 ± 6.1	132.4 ± 32.9	50.5 ± 14.8	136.6 ± 14.4	9.6 ± 3.0	156.4 ± 14.2	118.9 ± 11.8
Total mean	68.0 ± 16.9	26.3 ± 5.4	116.8 ± 17.7	45.4 ± 8.4	155.5 ± 14.0	14.5 ± 4.0	155.5 ± 11.2	88.4 ± 15.9
